# For more than love or money: attitudes of student and in-service health workers towards rural service in India

**DOI:** 10.1186/1478-4491-11-58

**Published:** 2013-11-21

**Authors:** Sudha Ramani, Krishna D Rao, Mandy Ryan, Marko Vujicic, Peter Berman

**Affiliations:** 1 Indian Institute of Public Health, Hyderabad, Plot # 1, A N V Arcade, Amar Co-operative Society, Kavuri Hills, Madhapur, Hyderabad 500 081, India; 2 Public Health Foundation of IndiaISID Campus, 4 Institutional Area, Vasant Kunj, New Delhi 110070, India; 3 Health Economics Research Unit, Division of Applied Health Sciences, College of Life Sciences and Medicine, University of Aberdeen, Foresterhill, Aberdeen AB25 2ZD, Scotland; 4 Human Development Network, The World Bank, 1818 H Street, NW, Washington DC 20433, USA; 5 Department of Global Health and Population, Harvard University, 665 Huntington Avenue, Building I, 11th Floor, Boston, Massachusetts 02115, USA

**Keywords:** Human resources, Incentives, Rural retention, Rural recruitment, Primary healthcare, India

## Abstract

**Background:**

While international literature on rural retention is expanding, there is a lack of research on relevant strategies from pluralistic healthcare environments such as India, where alternate medicine is an integral component of primary care. In such contexts, there is a constant tug of war in national policy on “*Which health worker is needed in rural areas?*” and “*Who can, realistically, be got there?”* In this article, we try to inform this debate by juxtaposing perspectives of three cadres involved in primary care in India—allopathic, ayurvedic and nursing—on rural service. We also identify key incentives for improved rural retention of these cadres.

**Methods:**

We present qualitative evidence from two states, Uttarakhand and Andhra Pradesh. Eighty-eight in-depth interviews with students and in-service personnel were conducted between January and July 2010. Generic thematic analysis techniques were employed, and the data were organized in a framework that clustered factors linked to rural service as organizational (salary, infrastructure, career) and contextual (housing, children’s development, safety).

**Results:**

Similar to other studies, we found that both pecuniary and non-pecuniary factors (salary, working conditions, children’s education, living conditions and safety) affect career preferences of health workers. For the allopathic cadre, rural primary care jobs commanded little respect; respondents from this cadre aimed to specialize and preferred private sector jobs. Offering preferential admission to specialist courses in exchange for a rural stint appears to be a powerful incentive for this cadre. In contrast, respondents from the Ayurvedic and nursing cadres favored public sector jobs even if this meant rural postings. For these two cadres, better salary, working and rural living conditions can increase recruitment.

**Conclusions:**

Rural retention strategies in India have predominantly concentrated on the allopathic cadre. Our study suggests incentivizing rural service for the nursing and Ayurvedic cadres is less challenging in comparison to the allopathic cadre. Hence, there is merit in strengthening rural incentive strategies for these two cadres also. In our study, we have developed a detailed framework of rural retention factors and used this for delineating India-specific recommendations. This framework can be adapted to other similar contexts to facilitate international cross-cadre comparisons.

## Background

The shortage of qualified health workers in rural areas has been recognized as a major impediment to the implementation of universal healthcare policies in many low- and middle-income countries [1-3]. India has been identified as one of the 57 countries across the globe with a substantial shortage of health workers [4]. Indeed, the density of qualified allopathic doctors, nurses and midwives is a fourth of the WHO benchmark of 2.5 per 1,000 population required for high coverage of health services in cross-country comparisons [5,6]. This shortage manifests itself even more starkly in the distribution of health workers servicing India’s rural areas. In 2005, for every 10,000 people, there were around ten qualified doctors in urban but only one in rural areas [6]. Among nurses, there is a three-fold difference in the density (per 10,000 population) of nurses and midwives between urban (15.9) and rural (4.1) areas of India [6]. Not surprisingly, unqualified practitioners have occupied the rural workforce space: national surveys indicate that up to 63% of clinicians practicing in rural India have inadequate training [6]. There has been much interest within the country in developing policy solutions to bring health workers to underserved areas, resulting in the launch of several governmental schemes.

Current strategies to address the shortage of rural health workers in India

1. Mandatory rural service: This involves making a stint of rural service mandatory for all allopathic graduates. This strategy has been attempted in several states; however, international evidence has shown that such schemes are often not well received [7,8]. 2. Incentives for rural service: This includes provision of higher salaries for rural service, rotational postings in rural areas, investment in infrastructure and logistics, and reserving seats for specialist training for in-service doctors who undertake some years of rural service. 3. Inclusion of Indian Systems of Medicine: In many states, clinicians trained in Indian systems of medicine (Ayurveda, Yoga, Unani, Siddha and homeopathy) —collectively known as AYUSH—have been posted in primary health centers (PHCs) to mainstream Indian systems of medicine [9]. 4. Non-clinician doctors: The Central Health Ministry in India, along with the Medical Council of India, is developing a new 3-year course, the Bachelor of Rural Health Care, graduates of which are meant to provide primary care in rural areas. There is some evidence from the experiments of Chhatisgarh, India, that this cadre is competent in managing conditions seen in primary care settings [10].

Despite such schemes, the question “*what do primary healthcare workers want?*” has seldom been asked. Specifically, voices of health workers (both those just entering service and those already in service) that should have been central to designing interventions for rural recruitment and retention have rarely entered the political domain in India.

In this article, we aim to

1. Explore the attitudes of students and in-service health workers from the allopathic, Ayurvedic and nursing cadres toward rural service.

2. Compare attitudes toward rural service of the three cadres.

3. Arrive at a generic framework that can facilitate the comparison of detailed rural recruitment themes across cadres.

A brief background of these three cadres studied is given below in Additional file 1.

### What this article adds

A vast expanse of global literature exists on the job choices of health workers. Surmising from these, financial incentives have been found to be important, but not adequate as an isolated measure for the uptake of rural jobs [11-13]. Career development and education opportunities in rural areas are important job determinants [14]; demotivation among health workers often stems from professional dissatisfaction [15]; health workers fear that prolonged rural postings may hinder career opportunities [16]. Further, health workers sought workforce management initiatives such as supervision and performance appraisals [17] as well as recognition/appreciation by seniors [18,19]. Prior exposure to rural areas and exposure to rural practice was found to aid rural retention [20]. Further, the social context such as security and safety of families has been shown to influence motivation [21]. Summarizing, several studies point out that the interaction between multiple factors such as career growth, organizational policies, working and living conditions play important roles in determining job location choices of health workers [22-26]. A recent review of these strategies in LMICs concludes that intervention strategies work better when ‘bundled’ together [27].

This article adds to existing studies in the following manner. First, contextual evidence from India on job choices of health workers has largely been piecemeal [28-30]; research that appreciates the nuances of factors that determine job choices has been sparse from this geographical area. There is little local evidence on this topic to inform national policy decisions. Second, the views of cadres that deal with alternate medicine (such as Ayurvedic doctors) have been solicited by few human resource studies worldwide, though such cadres constitute an important component of the pluralistic health workforce in many countries [5]. Yet, efforts to incentivize Ayurvedic doctors for rural care have been negligible. Third, this study attempts a qualitative comparison of the interests of the three principal cadres at a primary health center in India. Such comparisons are few in the current literature, and studies generally tend to focus on one cadre of human resources (usually general doctors or nurses).

## Methods

This is a qualitative study based on 88 in-depth interviews conducted with student and in-service allopathic doctors, ayurvedic doctors and nurses (Table 1).

**Table 1:** Selection of participants

**Categories**	**Total**
Allopathic students - undergraduate	23
Allopathic students - postgraduate	19
Ayurvedic students - undergraduate	8
Nursing students	12
In-service allopathic doctors	9
In-service Ayurvedic doctors	8
In-service nurses	9
**Total**	88

The study was conducted in two states in India: Andhra Pradesh (AP) and Uttarakhand (UK).

Both have shortages of health workers; AP is located in the southeast of India and is the fifth largest state in the country. UK is situated in the north and is a relatively small, mostly mountainous state. At the time of this study, AP had 36 medical schools and 206 nursing schools, while UK has few private schools and no government medical or nursing institutions.

We chose these two states purposively since we felt opinions of students/in-service health workers might differ in places that are geographically and demographically distinct (UK has a large mountainous terrain, which makes access difficult, so recruiting health workers might be more difficult in such places); also, perspectives of respondents might differ in places where the production capacity is different (AP having a large number of schools might have a larger pool of health workers available to the public sector).

### Selection of study participants

Participants included final year students and in-service persons from the allopathic, Ayurvedic doctors and nursing cadres. We used the principles of maximum diversity to choose our participants.

Student participants enabled us to explore factors affecting initial recruitment of health workers. A total of 62 students in the final year of their degree were interviewed from medicine, nursing and Ayurvedic medicine schools (Tables 1 and 2). These schools were purposively selected for diversity in terms of location as rural and urban, academic reputation and ownership (public/private), based on the following premises: rural schools are more likely to provide rural doctors [31]; students from institutes of higher academic repute might choose jobs differently from others; students from public institutes (where the fee is subsidized by the government) might have fewer financial obligations on completing their course and choose jobs differently (Table 2). The students belonging to the nursing subgroup and Ayurvedic subgroups were generally homogeneous in terms of age, background and gender (females in the case of nurses only), and variation in the types of schools was less (e.g., in the UK, there were no public schools for nurses). However, in the allopathic subgroup, there was more diversity in terms of age, numbers of schools, type and location of school, degree (undergraduate or post-graduate) and various specializations among post-graduate students. This resulted in a larger sample from the allopathic cadre. Within the schools, we asked school authorities to facilitate our interactions with students from both genders (except nurses, where participants were mostly female) and varied academic standings (good, average and poor as deemed by the school authorities). In the case of PG allopathic students, we also included different specializations as a selection factor.

**Table 2:** Distribution of institutes from which students were selected

**Andhra Pradesh**		
Hyderabad (state capital)	Allopathic	1 Public and 1 private school
	Nursing	1 Private school
	Ayurvedic	1 Public school
Kakinada (small town)	Allopathic	1 Public school
	Nursing	1 Public school
Uttarakhand*		
Dehradun (state capital)	Allopathic	1 Private school
	Nursing	1 Private school
	Ayurvedic	1 Private school
Haldwani (small town)	Allopathic	1 Public school

*There are no public nursing schools in Uttarakhand.

In-service participants (participants currently doing rural service in primary health centers) complemented the views of students by adding insights from the rural organizational setup and life in rural areas (Table 3). In each district in which the selected medical school was situated, one or two primary health centers (PHCs) around 50 km from the district center and having the full complement of staff in position, i.e., allopathic doctors, Ayurvedic doctors and staff nurses, were purposively selected (total, 8 PHCs). While this was a sample of rural PHCs, we did not cover extremely remote or inaccessible PHCs in this study; however, many of the staff in the PHCs we visited had some experience of postings in more difficult areas.

**Table 3:** Distribution of PHCs from which in-service personnel were selected

**State**	**District**	**PHC**
Andhra Pradesh	Rangareddy	2 PHCs
	Kakinada	2 PHCs
Uttarakhand	Dehradun	2 PHCs
	Haldwani	2 PHCs

### Data collection

Verbal consent was taken from all participants before the interviews^a^. Interviews lasted between 20–60 min and were primarily conducted in English, but where appropriate, the local languages of Hindi or Telugu were used. Interviews were audio-recorded and transcribed verbatim. Once the interviews had begun, several differences in perspectives among members of the three cadres emerged; hence, we aimed at obtaining data saturation within each cadre during data collection. Please refer Additional file 2 for details of the topic guide used for data collection.

### Data analysis

Data collection and analyses were done by the same team of six study investigators. Generic thematic analysis techniques [32-34] were employed, and the three concurrent steps of data analysis activity described were followed: data reduction, data display and conclusion drawing [33].

Information contained in the interviews was initially hand-coded line by line by the study investigators (open coding). For each of these interviews, a matrix was constructed compiling broad themes—from now on referred to as ‘factors’—mentioned by a respondent, its current level (what the situation is presently) and desired level (what would the respondent want it to be). Individual matrices were discussed in a team of 5–6 members. Through these deliberations, factors were defined in a standard manner and ordered hierarchically (divided into ‘major factors’ and ‘sub-factors’). Following this, the revised individual matrixes were compiled to generate a rough thematic framework.

**Figure 1:**
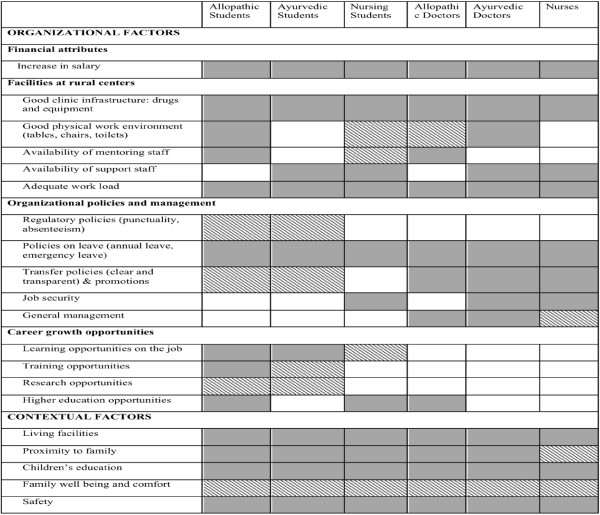
Factors that influence rural recruitment and retention.

This thematic framework was again applied to the data by the study investigators in teams of two. Through an iterative consultative process among the investigators, this framework was refined and the factors were re-arranged or re-defined. The final framework into which the factors were collated is given in Table 4. Using this framework, a comparative table was constructed that juxtaposes factors important to different cadres of respondents (Table 4). This table focused mainly on organizational and contextual components of the framework^b^. The initial version of this table had descriptive material (quotes and reflective remarks from researchers) as well as scores of numeric occurrence of a factor. This version was condensed through a consultative process using these criteria: a strong factor was one that was mentioned by the majority of the respondents in a cadre and not contradicted by other respondents (shown as a dark shading in Figure 1; a weak factor was either not widely encountered or qualified by other respondents from the cadre (shown as criss-cross shading in Figure 1). This method has been adapted from Miles and Huberman, 1994) [33]. This matrix has been used as the basis for discussion in this article.

**Table 4:** Final analytical framework for clustering factors affecting rural service

**Individual**	**Organizational**	**Contextual**
• Age	• **Financial attributes**	• Living facilities (housing, electricity, water, access to the market, hygiene)
• Gender	• Salary	• Proximity to family (near hometown)
• Marital status	• **Facilities**	• Children’s development (availability of good schooling, extra activities, future opportunities)
• Need for respect/self-esteem (recognition for work, sense of fulfillment, prestige of the job)	• Clinic infrastructure (drugs, equipment, laboratories, ambulance)	• Family's well-being and comfort (spouse job availability, spouse career growth, support to parents)
• Personal attitudes toward rural work	• Physical work environment (cleanliness, availability of water, electricity, toilets, good furniture, good construction, private cabins)	• Safety (physical security, legal protection against political interference)
• Familiarity with rural context	• Support staff (helping hands for working)	• Connectivity (transport availability, no sense of isolation)
	• Mentoring staff (for advising and guiding)	• Social life (entertainment facilities, social circle)
	• Workload (fixed working hours, shift systems, adequate number of patients)	• Community type (comfort and connect with the community, no language barriers)
	• **Organizational policies and management**	
	• Transfer policies and promotions (transparent policy, time of service in rural area clearly stated, no political interference in transfers)	
	• Job security (permanency of job, pensions)	
	• Regulatory policies to regulate absenteeism, punctuality of staff)	
	• Policies on leave (ability to take leave when required, especially emergency)	
	• Management (administration, bureaucracy)	
	• **Career growth opportunities**	
	• Learning opportunities on the job	
	• Training opportunities	
	• Research opportunities	
	• Postgraduate opportunities	

### Ethical approval

Ethical clearance for this study was obtained from the Institutional Review Board at the Public Health Foundation of India.

## Results

A brief profile of study participants is given in Table 5.

**Table 5:** Profile of student participants

	**Students**				**In-service personnel**		
	**Allopathic undergraduate**	**Allopathic postgraduate**	**Nursing**	**Ayurvedic**	**Allopathic doctor**	**Nurse**	**Ayurvedic doctor**
Number	23	19	12	8	9	9*	8**
Mean age in years	21.3 (0.9)	29.0 (5.1)	21.5 (1.3)	23.3 (1.5)	36.3 (3.8)***	29.8 (5.2)	29.6 (2.6)
Sex male (%)	52.1%	52.6%	8%	50%	66.6%	0%	16.6%
Studying in public institutes (%)	56.5%	57.8%	33.3%	50%			
Location of institute where studying	69.5%	63.1%	66.6%	100%			
Urban (%)							
Early schooling/ upbringing in urban areas (%)^#^					88.8%	85.1%	100%
Mean years of work experience					11.2 (3.3)	8.1 (5.2)	6.8 (3.0)

#Urban here includes cities and small towns.

*Two doctors’ profiles not available, **two nurses’ profile not available ***three data points missing.

Figure 1 presents a comparison of factors perceived to be important by students and in-service personnel from the three cadres. Certain organizational issues figure prominently: salary, the need for good clinical infrastructure and leave policies. In general, students placed more emphasis on career growth opportunities and the in-service personnel on organizational culture and management issues. Both groups emphasized concerns about contextual factors pertaining to living in a rural area. Table 6 is a summarized version of Figure 1.

**Table 6:** Key differences among the three cadres of health workers

**Students**	**Allopathic cadre**	**Ayurvedic cadre**	**Nursing cadre**
**Attitude toward rural primary care**	● → Preferred jobs were in the urban private sector	● → Open to both private and public job options, though preference was for the public sector	● → Generally preferred jobs in the public sector. A few nurses had ambitions to work abroad
	● → Rural primary care jobs are perceived as not providing professional growth and respect	● → Jobs in the primary health center give legitimacy to alternate health professions	
			● → Rural primary care jobs in the public sector offered job security and satisfying work hours
	● → Specialization/postgraduate opportunities given utmost importance		
**Key concerns of students in taking up rural primary care jobs**	● → Lack of professional growth	● → Poor salary	● → Job security wanted
		● → Inability to practice alternate medicine	
			● → Adequate workload
	● → Poor salary	● → Lack of work related facilities	● → And regular timings wanted
		● → Several contextual factors	
	● → Lack of job prestige		● → Inability to take leave during emergencies
			● → Poor personal security
	● → Lack of work-related facilities		● → Several contextual factors
	● → Several contextual factors		
**Concerns of in-service personnel in rural primary care jobs**	● → Fear of prolonged stints with no guarantee of transfers from rural area	● → Fear of prolonged stints with no guarantee of transfers from rural area	● → Fear of prolonged stints with no guarantee of transfers from rural area
		● → Poor status and salary in comparison with allopathic doctors	
			● → Inability to take leave
			● → Inability to assist patients during the
	● → Poor salary compared to private practitioners		
			● → Several contextual factors
		● → Lack of Ayurvedic drugs	
		● → Several contextual factors	
	● → Bureaucracy in public sectors jobs		
	● → Several contextual factors		

The important findings from our analyses are discussed below:

### Organizational factors

#### Salary

Salary was cited as an important concern in selecting a job, but its significance varied among the cadres (refer Additional file 3). Respondents from the allopathic cadre felt that their general financial remuneration in the market was inadequate compared to the effort put into becoming a doctor and what their compatriots in other equivalent professions such as engineering earned. Additionally, they acknowledged that public sector jobs were less lucrative than private options in urban areas. There was a clear preference for private sector jobs in this cadre. Ayurvedic respondents felt that differences in terms of earning between the public and private sector were considerable; however, public sector jobs offered them a professional legitimacy and increased their chances of earning better through private practice in the future. Many in this cadre were unsatisfied with their current levels of salaries and with the fact that their earnings were lower than those of allopathic doctors. To this cadre, salary appeared to be important in terms of giving them professional parity with allopathic doctors.

Unlike the other two cadres, respondents from the nursing cadre were generally satisfied with the current level of salaries in the market. For nurses, both the public and private sectors offered almost equal remuneration. Respondents from the nursing cadre acknowledged that, while financial incentives were important in their job choice decisions, they were willing to take up rural jobs without specific monetary inducement (especially if the job was with the public sector and offered security in lieu). Interestingly, an attitude of service was often reflected in conversations with nurses, which we did not come across in the other two cadres^c^.

Respondents from all three cadres acknowledged that while financial incentives were important, these were not adequate to attract or retain them in rural jobs. Many in the allopathic cadre were willing to forgo rural incentives—even increases in salaries 30% to 50% of current levels—if the job came in the way of their postgraduate education. Across all cadres, when salary was considered in the context of a rural job, its salience diminished. Respondents explicitly state that life in a rural area—if taken up for the sake of financial incentives alone—has repercussions in terms of affecting children’s schooling, and involved a sacrifice of personal and family life.

#### Facilities at rural health centers

Perceptions of work facilities in rural areas were important in determining job preferences across cadres (refer Additional file 4). Concerns about good clinical infrastructure—equipment, drugs, ambulance, operation theatre facilities, sterilization and testing equipment, and basic laboratory test kits—were frequently emphasized by respondents in all three cadres. Lacks in clinical infrastructure made respondents across cadres feel limited in their capacity to use their skills and serve the community. Among Ayurvedic doctors, one specific concern was the lack of Ayurvedic medicines at PHCs, which inhibited their ability to practice their system of medicine. In addition to good clinical infrastructure, allopathic students and some nursing students mentioned the need for a good physical work environment at PHCs—such as having basic infrastructure like tables, chairs and toilets with running water. Some Ayurvedic doctors felt that better infrastructure (rooms, desks, air conditioning) was given to allopathic doctors even within the same PHC; this discrimination made them feel de-motivated^d^.

The lack of mentoring staff was mentioned mostly by respondents in the allopathic cadre—by both students and younger in-service doctors. These respondents were wary of making mistakes in diagnoses and treatment with no colleagues or specialists to guide them in remote areas. Mentoring was not perceived as a significant concern in the other two cadres. Nurse respondents looked upon doctors as mentors and were happy to serve in PHCs as long as doctors were available. However, the availability of support staff was often mentioned as an important concern by respondents of the nursing and Ayurvedic cadre. Nurses felt that the major burden of all duties, including handling medication, cleaning and other administrative jobs, fell on them in the absence of other staff to take on these responsibilities. Among Ayurvedic respondents, there was a demand for support staff such as special compounders who would assist them in preparation of medication.

### Organizational policies and management

Respondents across cadres emphasized several organizational policies and management issues that were important to their functioning in a rural job. In-service respondents were more emphatic in mentioning this category of factors.

Respondents across cadres felt that their ability to take leave was restricted in public sector rural jobs. Many respondents felt that since rural jobs potentially required them to stay away from families, additional leave to visit family must be given. Difficulty in taking leave during rural stints seemed to be rooted in the fact that substitute health workers were often not available. Also, it was mentioned that leave applications in the public sector generally required time to be processed, and sudden leaves during emergency were disapproved. A difficulty in getting formal leave appears to be a potential contributor to increasing unofficial leave and absenteeism at PHCs.

Another organizational issue often raised by respondents was the lack of a fair system of “rotation” or transfer within the public sector. The current system of transfers within the public sector is state-specific (and often not clearly specified through guidelines); transfers are generally indicated on two occasions—on request of the candidate or when a candidate has completed the maximum years permissible in one stint. A large number of transfer requests are usually made to more desirable posts (urban or near urban posts); hence, not all requests are entertained by the government. In addition, once a candidate settles in a desirable post, there is no intention on his or her part to move. Many candidates felt that both processes—getting to a desirable post and being stationed at a desirable post for a long time—required political clout. Due to lack of specific guidelines, some health workers could escape rural stints as well as stay at desirable posts for durations longer than specified. Health workers without political influence feared being stuck in remote areas for long periods of time, being unnoticed and not being considered for promotions.

The importance of having a fair transfer policy played out strongly in interviews with in-service allopathic doctors and nurse. . Policies that were transparent and specific about duration and place of posting were often demanded. Indeed, given the importance given by in-service respondents to transfer policies, it is remarkable that many states in India do not have a transfer policy that guarantees that workers will be rotated between hardship and non-hardship posts (refer Additional file 5).

#### Job security: why nurses and Ayurvedic doctors preferred a public sector job

Respondents from the allopathic cadre felt that urban private sector jobs paid better and indicated a clear preference for these jobs. However, respondents from the nursing and Ayurvedic cadre demonstrated a preference for public sector (government) jobs*.*

For nurses, salaries were almost equal in both public and private sectors. Most nurses seem to prefer public sector jobs because of job security and better work timing. (Among the participants, there were two nurses from an urban private nursing school that preferred to work abroad because of better career prospects and financial considerations. However, they were not opposed to government jobs either and regarded these with equal respect). For the sake of a public sector job, nurses were often willing to put up with rural stints. For Ayurvedic respondents, both job security and the potential to establish themselves as primary care practitioners made jobs at PHCs valuable. Both nurses and Ayurvedic doctors felt that the demand for public sector jobs in their cadres was such that if a candidate refused a rural stint, his job would be at stake since many others would be willing to accept the post. This attitude of respondents from the Ayurvedic and nursing cadres toward public sector jobs is noteworthy—while this does not mean that they were always favorably disposed toward working in rural areas, they do constitute an available and qualified workforce that can be incentivized to serve in rural areas through government jobs.

#### Career growth

Among allopathic students, becoming a specialist was highly prized. All allopathic students interviewed aspired to enroll in a postgraduate course after completing their undergraduate degree (MBBS). This was universally seen as a necessity for career progression—across allopathic students of different sexes, different backgrounds and school types. Consequently, allopathic students were often not interested in entering the job market after finishing their undergraduate degree. Interestingly, financial benefits were not perceived to be the principal motivation behind specialist training. However, respondents did acknowledge that as specialists, better salaries could be expected. It was also felt that a PG degree aided private practice, since patients often preferred to go to doctors with a specialization.

The key ideas associated with PG education appear to be prestige and a chance to have a more professionally challenging career. The desire to become a specialist and to have the professional life it offered are also closely linked to the way primary care is currently viewed by the medical profession. A job in primary care is often looked down upon as an inferior alternative and is regarded with less respect. Respondents felt that working in a rural PHC was not as prestigious as working in an urban tertiary health facility. In addition, many allopathic students were concerned that lack of focus on practical skills during the undergraduate training did not equip them to perform clinical tasks well. Further, undergraduate training provided students with very limited exposure to health problems of rural areas. As a result, these students expressed their unwillingness to man rural PHCs independently. Undergraduate students also felt that at PHCs, their exposure to ailments—other than basic illnesses—would be limited, and this would impact their learning. On the other hand, postgraduate students felt that they were overqualified to work in a primary health center (typically the first posting for a doctor joining government service). They expressed a preference for tertiary care roles where they felt their knowledge could be put to better use.

This emphasis on postgraduate education and a disinclination to work in primary care was unique to the allopathic cadre (both students and in-service doctors). Some nursing students did express an interest in doing a postgraduate course or in upgrading their current undergraduate degree (upgrading a general nursing degree of 3.5 years to a bachelor’s degree of 4.5 years). However, this was not considered as essential to their career progression. Ayurvedic students generally emphasized “on-the-job” learning rather than postgraduate education. Unlike the allopathic cadre, respondents from the Ayurvedic and the nursing cadres often looked upon jobs at PHCs as good training grounds that would better their careers (Refer Additional file 6).

### Contextual factors

The living environment of rural India was an important area of concern across cadres. The availability of good living facilities in rural areas—including good housing, availability of electricity and water—was cited as an important consideration while taking up a rural job. In-service respondents felt that even if the government provided accommodation in rural areas, these houses were often decrepit and lacked maintenance and running water. Quarters within the premises of the health center or close by, with round-the-clock water and electricity, made of cement and having toilets were considered as basic necessities for a “good standard of living,” and these were demanded. Some respondents were willing to travel to rural PHCs from nearby areas where good housing was available (so that their family could stay there comfortably). However, the availability of transportation was also mentioned as an important concern; related issues included infrequent public transportation (few public buses), bad roads and poor connectivity. (Refer Additional file 7).

The education of one’s children appeared to be one of the key factors affecting job choices. Respondents across cadres emphasized that rural schools had poor-quality teachers, poor infrastructure, did not teach English and did not provide a healthy environment for children’s development. All respondents wanted their children to study in the best private schools where English was the medium of instruction so as to secure a better future for them. It was also felt that children growing up in urban areas had better exposure and better future prospects. Some respondents were willing to take up rural stints before their children started formal schooling but not after. Some respondents were willing to send their children to good private schools in the city if the government would bear the cost while they served in rural areas. However, many respondents were also disinclined to live separately from family on a long-term basis. Some respondents also mentioned the need to stay with elderly parents who needed medical care in the city.

Living in rural areas was also associated with a lack of social life, especially by respondents who were used to an urban lifestyle. Many students felt that rural areas lacked a peer group and entertainment facilities (going to restaurants and malls, watching movies). Allopathic students underscored that the “type of community” they would have to live in would be an important determinant of job choices they make: some students felt strongly that they did not have the ability to blend into rural life and establish a rapport with a rural community (which might be illiterate and also speak a different language). However, we did encounter one allopathic student from a rural background, whose views deviated from the rest of the cadre; he expressed an inclination to work in rural regions:

Because I come from a small village, (I know that) there are no good facilities there for health care. I am very much concerned about those health facilities. (Allopathic student, male, post graduate, rural public school, AP)

But this viewpoint was an exception.

Another important issue was safety (also termed “security” by some respondents). Female respondents specifically emphasized the importance of timely transportation and the need for colleagues and security guards at the workplace who could assist them in case of brawls within the PHC premises. Many respondents reported incidents—either experienced by them or hearsay—about staff in the PHC being blamed for poor medical practices and hence being physically assaulted by members of the community.

It was interesting to note that contextual factors were more salient for students than for in-service personnel—this was probably due the fact that the majority of students were not familiar with work in rural settings. No major differences among the cadres could be observed with regard to this range of factors.

## Discussion

Our study is based on the perspectives of students and in-service respondents from various cadres and has its limitations. For one, the student respondents (especially those in the allopathic cadre) had not given serious thought to entering the job market since they were interested in pursuing further training. This limited their understanding of the job market. Second, in-service respondents selected in this study worked at rural centers that were not distant from urban centers. Consequently, the views of these respondents could be different from those who serve in more remote areas. Lastly, this is a qualitative study, and we did not attempt to prioritize one attribute over the other; rather, we have tried to “bundle” together the key concerns of students and in-service health workers and compared these concerns across the three cadres (Table 6).

The findings reported in this study portray a large number of factors that affect job preferences of students and in-service health workers. This multiplicity suggests that “packages” of pecuniary and non-pecuniary incentives rather than singular incentives, such as current strategies of offering only higher salary, will have a greater effect on rural recruitment. Broad scrutiny of these factors indicates that these packages can include an increase in salary, opportunities for career growth, better living conditions for the families, better equipped health facilities and improved organizational support policies. Many of the factors elucidated in this study are not new and confirm findings from other international studies discussed earlier [15,16,18,23]. These factors, however, have not been studied in the Indian context. Also, there have been few attempts to organize these factors within a framework. For instance, Lehmann et al. employ a broad model for their systematic review consisting of individual, organizational, local, national and international contexts [27]. Schoo et al. (Australia) and Cameron et al. (Canada, doctors only model) presented detailed elements of retention within organized domains [35,36]. However, we did not come across frameworks in the literature that could facilitate the comparison of detailed themes across cadres and from a developing country context. One contribution of our study is the visual framing of the organizational and contextual factors that emerged in a systematic manner. This visual framing makes the derivation of policy lessons easier.

In this study, it was found that the professional and personal ambition of allopathic students was not compatible with being stationed at rural PHCs on a long-term basis. Urban private employment was preferred over public sector jobs for the following reasons: these were more lucrative, offered better facilities, working environment and mentorship, and were considered more prestigious. Moreover, primary care jobs anywhere were viewed as a hindrance to professional growth, having inadequate mentorship and offering few chances for having a professionally challenging career. Many were willing to forgo any amount of rural financial incentives if the job came in the way of their postgraduate education. However, many allopathic students were willing to take up a rural stint in exchange for a PG-related incentive. For this cadre, becoming a specialist was highly desirable.

This observation corroborates evidence from other country contexts where decreased interest of allopathic doctors in primary care has been observed [37,38]. This viewpoint of the allopathic cadre has several implications. For one, it explains why better salary or many of the non-monetary incentives did not emerge as imperative for undertaking rural service among allopathic students. Moreover, the desire to train further and become specialists reduces the stock of allopathic graduates interested in entering government service and serving in primary health centers (PHC). Once they become specialists, they are both overqualified to work at a PHC and have an even lower inclination to serve in primary care rural jobs. However, the desire for specialization combined with the difficulty in getting admission to these courses can serve as an important ‘hook’ for rural service. Indeed, several states in India have utilized this ‘hook’ by offering preferential admission to specialist courses in return for a few years of rural service [39].

The recruitment of Ayurvedic doctors in government health services was initiated 5 years ago by the health ministry in India with intentions to mainstream traditional systems of medicine [9]. From the accounts of many in the Ayurvedic cadre, it is clear that government service is highly sought after. To this cadre, government jobs offered security of employment and a perceived legitimacy as professional doctors and aided in the establishment of a medical practice. Job characteristics important to the Ayurvedic cadre include salary, professional support systems and the availability of drugs. Salary was important to this cadre for two reasons—one, being contractual employees they are considerably underpaid compared to the allopathic cadre; second, higher salary would bring them greater parity with the allopathic cadre.

For the nursing cadre, too, government jobs meant stability and better work hours; thus, these jobs were held in high regard. For this cadre, rural service was particularly attractive if there was better financial compensation, an adequate workload and regular work hours, ability to take leave, and accommodation and postings were close to their hometown. Some states in India offer monetary incentives to nurses [30], and some, like Andhra Pradesh, offer a chance to upgrade the general nursing degree to a bachelor’s degree equivalent. While there is some evidence that these incentives work in international settings [11], little is known from the Indian context on the effect of these incentives on nurses. In our study, an attitude of service was strong in the nursing cadre (unlike the other two cadres), and many were often willing to take rural jobs without financial inducement. The perspectives of nurses in our study provide reason to consider incentives that encompass ideas of adequate leave, staff support and postings close to hometowns in addition to financial and career incentives (Additional file 8).

## Conclusions

The public sector in India has instituted several mechanisms, pecuniary and non-pecuniary, to attract health workers, particularly allopathic doctors, to rural areas. However, current mechanisms focus on singular issues (such as increasing salary). Our findings call for replacing this isolated-incentive approach by a ‘package’ approach. In India, for government efforts to place qualified health workers in rural health centers to be successful, it is clear that two conditions need to be fulfilled—both government service and rural postings need to be attractive to health workers. The first is necessary for bringing health workers into the public sector system and the second to motivate in-service health workers to remain in rural areas. The study findings indicate that attitudes toward both these conditions differ among the allopathic, Ayurvedic and nursing cadres.

Most respondents from the allopathic cadre did not find public sector service or working in rural primary care settings enticing. Our findings suggest that, in India, the “hook” of PG education incentives can be considered the most powerful mechanism to bring allopathic physicians to rural areas—on a temporary basis. Consequently, simply increasing the monetary incentives for rural service will do little to increase the presence of government doctors in rural areas in the long run. A much deeper and broader strategy, elements of which involve improving the general prestige of public sector service in the cadre, is required. In contrast, public sector service is prized in the nursing and Ayurvedic cadre. Incentivizing rural service for these cadres seems less challenging in comparison to that of the allopathic cadre; however, currently, the public sector in India offers few rural incentives for these two cadres. There is merit in strengthening rural retention strategies for the Ayurvedic and nursing cadres so as to bolster their role in rural primary care.

## Endnotes

^a^While written consent is the norm, personnel employed with the public sector in our study setting often demonstrate a reluctance to sign documents. To keep the consent process uniform, we used a verbal consent process (which was recorded) for all participants in the study.

^b^Some individual factors such as age and intrinsic motivation of a candidate to work in rural areas were mentioned as affecting the uptake of rural service in some interviews. But our data on these factors were weak, and we couldn’t piece together how exactly these factors affected rural service. Therefore, we retained this group of factors in the basic framework, but concentrated on organizational and contextual factors in further analyses.

^c^We could not find a direct justification for this marked difference in attitude among nurses (compared to the other cadres), but one explanation could be that since early nursing institutes in India were mainly run by Christian missionaries, the population selecting into the nursing profession is attuned toward service.

^d^Not much academic work has been done on the issues of Ayurvedic doctors working at PHCs. One other study briefly reports dissatisfaction among Ayurvedic doctors in AP due to differential allocation of infrastructure: Lakshmi JK: Less equal than others? Experiences of AYUSH medical officers in primary health centres in Andhra Pradesh. *Indian J Med Ethics.* 2012, 9:18–21.

## Abbreviations

AP: Andhra Pradesh; AYUSH: Ayurveda yoga, Unani, Siddha and homeopathy; BAMS: Bachelor in Ayurvedic medicine and surgery; GNM: General nursing and midwifery; MBBS: Bachelor of bedicine and bachelor of surgery; PG: Postgraduate; PHC: Primary health center; UK: Uttarakhand.

## Competing interests

The authors declare that they have no competing interests.

## Authors’ contributions

SR, KR, MV, MR and PB were involved in conceptualizing the study and designing the tools. Data were collected in a team that included SR and KR. SR and KR wrote the first draft of the manuscript, which has been reviewed and revised by MV, MR and PB. All authors read and approved the final manuscript.

## Supplementary Material

Additional file 1 Human resources for health in India: The allopathic, AYUSH and nursing cadres. Click here for file

Additional file 2 Description of the study sample and the topic guide. Click here for file

Additional file 3 Salary. Click here for file

Additional file 4 Facilities in rural areas. Click here for file

Additional file 5 Organizational policies and management. Click here for file

Additional file 6 Career growth. Click here for file

Additional file 7 Contextual factors. Click here for file

Additional file 8 Way Forward. Click here for file
