# Supplementary material for: For more than love or money: attitudes of student and in-service health workers towards rural service in India
Source: Hum Resour Health. 2013 Nov 21;11:58. doi: 10.1186/1478-4491-11-58 (PMC4222605; doi:10.1186/1478-4491-11-58)
Supplement: Additional file 1 — Human resources for health in India: The allopathic, AYUSH and nursing cadres. [file 1478-4491-11-58-S1.pdf]

Additional file 1

Human resources for health in India: The allopathic, AYUSH and nursing cadres

India has a diverse health workforce. According to the National Occupation Classification, providers of allopathic health services include doctors (general and specialists), dentists, nurses, midwives, pharmacists, technicians, optometrists, physiotherapists, nutritionists, sanitarians and other support staff. Doctors trained in Indian systems of medicine—Ayurveda, Yoga, Unani, Siddha —and Homeopathy, known as AYUSH, also provide health care. In addition, persons who have no formal qualification to practice medicine also do so, though their numbers are uncertain. In this study, we concentrate on three principal clinical care providers in India: allopathic doctors, AYUSH doctors and nurses. Allopathic doctors comprise 31% of the workforce in India, followed by nurses and midwives (30%). AYUSH practitioners constitute around 9% of the workforce.

Allopathic doctors are medical graduates with an undergraduate MBBS degree (5½-year duration including a year of internship) or postgraduate specialist diploma or degree registered with the Indian Medical Council. There are approximately 3.8 doctors per 10,000 population. Further, there are 11.3 (1.2) doctors per 10,000 population in urban (rural) areas. Around 80% of allopathic doctors are employed in the private sector in India.

Nurses are diploma holders in General Nursing and Midwifery (3½-year course) or those with a 4-year bachelor's degree or a 2-3-year post-graduate degree registered with the Indian Nursing Council. There are approximately 1.7 (4.3 in urban and 0.7 in rural) nurses per 10,000 population. About half the total nurses are employed in the private sector in India.

AYUSH graduates have a degree (5½ years including a year of internship) or postgraduate degree in Ayurveda, Unani, Siddha or homeopathy registered with the Central Council for Indian Medicine or the Central Council for Homeopathy. There are approximately 1.02 AYUSH doctors per 10,000 population, also concentrated in urban areas. About 80% of AYUSH doctors are employed in the private sector.

Adapted from: Rao KD, Bhatnagar A, Berman P: **So many, yet few: Human resources for health in India.** *Hum Resour Health* 2012, **10:**19
