# Supplementary material for: For more than love or money: attitudes of student and in-service health workers towards rural service in India
Source: Hum Resour Health. 2013 Nov 21;11:58. doi: 10.1186/1478-4491-11-58 (PMC4222605; doi:10.1186/1478-4491-11-58)
Supplement: Additional file 2 — Description of the study sample and the topic guide. [file 1478-4491-11-58-S2.pdf]

Additional file 2

Description of the study sample and the topic guide

Allopathic students: These are final year undergraduate allopathic students studying for the Bachelor of Medicine and Bachelor of Surgery (MBBS) degree. Postgraduate students were in the process of obtaining a specialist degree—obtained after 3 years of training in a specific medical specialty.

Ayurvedic students: The final year students were studying for the undergraduate Bachelor in Ayurvedic Medicine and Surgery (BAMS) degree that they receive after completing 5.5 years of training.

Nursing students: The final year students were studying for the General Nursing and Midwifery (GNM) degree, which they receive after 3.5 years of training.

In-service health workers: All the sampled in-service health workers were based at primary health centers (PHC) around 50 km from the nearest town. Allopathic doctors had an MBBS degree, the Ayurvedic doctors a BAMS degree and nurses a GNM degree.

Topic guide: Students

- Background
- Career objectives
- Plans after completion of course work
- Expectations from future jobs
- Specific rural urban preferences
- Perceptions of rural employments
- Policy options and incentives that appear appealing

Topic guide: In-service participants

- Background
- Experiences with current job
- Perceptions on employment in the rural public sector
- Future plans for careers
- Attitudes toward retention
- Discussion on policy options favoring retention
