# Supplementary material for: For more than love or money: attitudes of student and in-service health workers towards rural service in India
Source: Hum Resour Health. 2013 Nov 21;11:58. doi: 10.1186/1478-4491-11-58 (PMC4222605; doi:10.1186/1478-4491-11-58)
Supplement: Additional file 3 — Salary. [file 1478-4491-11-58-S3.pdf]

Additional file 3

Salary

Current level of salaries in the market for each cadre (as reported by respondents)

1 USD = 50 INR (approximately)

Allopathic cadre: INR 30,000-80,000

Ayurvedic cadre: INR 15,000-50,000

Nursing cadre: INR 10,000-30,000

(*For the allopathic cadre, higher salary levels cited were linked to private sector jobs; for the Ayurvedic and nursing cadres, no specific differences were cited)*

Allopathic cadre: Public sector jobs do not pay adequately

"It is very difficult to manage with this amount (in public sector jobs) after several years of study. Even after a diploma, the private sector offers more." (Allopathic student, 22, female, undergraduate, urban public school, AP)

"*If I compare myself with my engineer friends who were mediocre in studies…whereas I was good…now these people are financially settled." (Allopathic student, 23, male, undergraduate, rural public school, UK)*

"*Today it has become more commercial—most of the doctors are also thinking that if we go here (public sector) we get only rupees* 10,000 a month but if we go in private even for an hour we might get rupees 5,000-6,000—so probably shifting to that side is better.*" (*Allopathic student, 25, female, postgraduate, urban private school, AP)*

Ayurvedic cadre: Salary parity with allopathic doctors

"We should get salary at par (with MBBS)…yes, sir…we should not feel bad that we are getting less than others when our timings and work are the same." (Ayurvedic doctor, female, experience: 6 years, upbringing: city, UK)

"*Government has decided on a pay scale according to different 'pathys'…different for allopathy and Ayurveda. Ayurveda students think that 'we also study for 5½ years—same as MBBS students—and we also have to study allopathy. Then why is there is difference in pay scale?' We also spend same time on education and our degree is also approved by national councils." (Ayurvedic student, 21, male, urban private school, Uttarakhand)*

Nursing cadre: An attitude of service

"*We share the happiness and sorrows of all…We are serving and also being paid for it. My necessities are being fulfilled and I am able to serve*

others."(Nurse, female, experience: 14 years, upbringing: city, UK)

"*It is not about money, it is our duty to help. Our first aim is to help people."* (*Nursing student, 23, male, urban private school, AP*)

The rural context and financial incentives

"I may consider a rural area independently on a financial basis if I get 1.5 lakh (rupees)…but that is not the only issue, my kids won't get schools over there and if I have to send them boarding schools, then I know I will (be) sacrificing my personal life." (Allopathic student, 28 years, male, postgraduate, urban private school, UK)

"(Even if you provide desirable salary)…we have family problems. How can we stay (in a rural area)? Where would my child go for schooling?" (Ayurvedic doctor, female, experience: 6 years, upbringing: city, UK)

"Taking rural jobs (for extra pay) depends on the mentality of individual persons——some people work for money but some work for a better future." (Nursing student, 22, female, urban private school, UK)

**Rupees = INR*
