# Supplementary material for: For more than love or money: attitudes of student and in-service health workers towards rural service in India
Source: Hum Resour Health. 2013 Nov 21;11:58. doi: 10.1186/1478-4491-11-58 (PMC4222605; doi:10.1186/1478-4491-11-58)
Supplement: Additional file 4 — Facilities in rural areas. [file 1478-4491-11-58-S4.pdf]

Additional file 4

Facilities in rural areas

Clinical infrastructure

"In PHCs, we are expected to do lot of things in minimum possible facilities. Only if I am given a handful of things like a pharmacist, drugs, ambulance, equipment and all health care facilities I need –with which I can feel confident that I can help people—only then will I join a PHC." (*Allopathic student, 23, male, undergraduate, rural public school, UK*)

"(Though) I am a gynecologist, because I am posted at a PHC, I am limited to normal deliveries only…any complicated cases, I have to refer because I don't have an operating theater." (Allopathic doctor, female, experience: 8 years, upbringing: city, UK)

"In PHCs, they must provide us with casualty (wards), operation theatre, equipment, sterilization, 24 h services and a special ward for gynecology." (Nursing student, 20, female, rural public school, AP)

"For (conducting) deliveries, we need electricity back-up. Electricity may go at any point of time. Sometimes we have to conduct deliveries under candle light." (Nurse, female, experience: 1 year, upbringing: city, UK)

"Our supply of medicines is not enough. We need many different drugs. But these are not provided here." (Ayurvedic doctor, female, experience: 12 years, upbringing: city, AP)

"I think that MBBS doctors get space to do research. They have better instruments, laboratories, etc. He (the Ayurveda graduate) won't get lab, facilities with which he can conduct his research."(Ayurvedic student, 21, female, urban private school, UK)

Physical work environment

"The (work) place should be well furnished. I should have my own room…(in rural areas); you may not even have a desk. Such places are not that alluring." (Allopathic student, 22, female, undergraduate,

urban private school, UK)

Need for mentoring staff

"We want one senior person, like PGs or at least assistant professors…but they have to stay with us. They have to share their knowledge with us and then only (we can work). Because here when we have any problem, we approach our seniors and professors, to solve the problem…there (in rural areas) we have to lead an independent life…so if any problem arises…what will we do?" (Allopathic student, 25, female, undergraduate, urban public school, AP)

Need for support staff

"*Work force is also not adequate, in a rural area a nurse sometimes has to do the work of self, a ward boy and doctor too…so it is very difficult for us to do such a job." (Nursing student, 21, urban private school, UK)*

"Initially I was posted alone in AYUSH department. I was not having any pharmacist, ward boy, compounder…So I alone struggled and brought medicines from the (central) office and established the clinic, saw patients, dispensed medicines…" (Ayurvedic doctor, male, experience: 10 years, upbringing: city, UK)
