# Supplementary material for: For more than love or money: attitudes of student and in-service health workers towards rural service in India
Source: Hum Resour Health. 2013 Nov 21;11:58. doi: 10.1186/1478-4491-11-58 (PMC4222605; doi:10.1186/1478-4491-11-58)
Supplement: Additional file 5 — Organizational policies and management. [file 1478-4491-11-58-S5.pdf]

Additional file 5

Organizational policies and management

Leaves

"*Currently we don't have leaves——only 14 days annual leave. Sometimes during emergencies, our salaries also get deducted. At least we should have casual leave (that we can take) if we will get ill." (Nurse, female, experience: 5 year, upbringing: small town, UK)*

"I have heard that in emergency also if you apply it takes several days to get approved and sometimes it won't get approved at all. In emergency also they provide only 2-day leaves." (Ayurvedic student, 22, female, urban private school, UK)

"We should be given 3-5 days leave per month to visit home——otherwise weekends must be off."

(Nursing student, 22, male, urban private school, AP)

"I could only visit my family only on weekends…1½ to 2 days I would travel home…stay and go back…and other holidays also…for a family man, this is important."
(Allopathic doctor, male, experience: 11 years, upbringing: city, AP)

Transfer policies
"(The government) must place new doctors at remote PHCs and give them the assurance that they will be transferred after a definite period…and place experienced /senior doctors at headquarters." (Allopathic doctor, female, experience: 14 years, upbringing: city, UK)

"*I didn't get (a post at) the nearby PHC and I could not spend money. Some bribe we have to give, of course sir, for the post. I could not afford it and there was no vacancy there. Every year I applied for transfer. But I didn't get the job.*" *(Allopathic doctor, male, experience: 9.5 years, upbringing: city, AP)*

"At the time of joining they should tell us for how much time we will be posted in a particular place. Then we will definitely go. If there are rules on board, then it is fine." (Nurse, female, experience: 20 years, upbringing: village, UK)

"If you will ask me to serve in interior for 3 years and if my family is staying in a (safe) campus, then I don't have any problem. But the government should exactly tell me that for 3 years, you would be there…and next posting, we will give you in a city or better place." (Allopathic doctor, male, experience: 12 years, upbringing: city, AP)

Job security: Preference for government jobs in the Ayurvedic and nursing cadres

'The main thing is I want to get a government job whether it is in a rural area or it is in an urban area. In private hospitals, there will be lot of work…24 h…and the job is not permanent." (Nursing student, 21, female, rural public school, AP)

'I used to work in a private job…but we had to do 24 h duty…sometimes I had to do triple shifts…I used to get tired." (Nurse, female, experience: 14 years, upbringing: city, UK).

"Any job is okay… I prefer government since I can practice privately anyway. Because my ultimate aim is to settle down in a good position."

(Ayurvedic student, 22, male, urban public school, AP)

"I am on contract. I came to work here due to government's tag—there is no other real benefit. A government job has certainty. I was insecure in my previous job." (Ayurvedic doctor, male, experience: 9 years, upbringing: small town, UK)

"Government job means security. Your name builds up. Once your name builds up then it (private practice) runs." (Ayurvedic doctor, female, experience: 6 years, upbringing: city, UK)

Preference for private practice in the allopathic cadre

"*Whatever you do here, (the) ultimate for any doctor is his own clinic. I don't know what it is but it is a thing that comes from inside." (Allopathic student, 22, male, undergraduate, urban private school, UK)*

"*A few years in the government in okay…but ultimately I want to move to the private (sector)." (Allopathic student, 22, male, undergraduate, urban private school, AP)*

Today it has become more commercial—most of the doctors are also thinking that if we go here (public sector) we get only Rs 10,000 a month, but if we go in private even for an hour we might get Rs 5,000-6,000—so probably shifting to that side is better." (*Allopathic student, 25, female, post-graduate, urban private school, AP)*
