# Supplementary material for: For more than love or money: attitudes of student and in-service health workers towards rural service in India
Source: Hum Resour Health. 2013 Nov 21;11:58. doi: 10.1186/1478-4491-11-58 (PMC4222605; doi:10.1186/1478-4491-11-58)
Supplement: Additional file 6 — Career growth. [file 1478-4491-11-58-S6.pdf]

Additional file 6

**Career growth**

**Postgraduation as a natural career progression for the allopathic cadre**

*"I have not really thought of any job after this course…only of institutes to which I can apply for PG."* (*Allopathic student, female, undergraduate, urban private school, AP*)

*"No MBBS person will like to settle with only an MBBS. There is no term like a general doctor or a general surgeon, everything is broken now, everything is specialized…" (Allopathic student, 27, male, post graduate, urban public school, AP)*

*"Society doesn't recognize an MBBS. Obviously if you have money, you would not like to go to a less qualified doctor."(Allopathic student, 22, female, urban private school, UK)*

*"As a simple MBBS doctor without PG degree, you don't get much options in city and being a plain MBBS doctor, if you work in city you don't have much status." (Allopathic student, 25, female, postgraduate, urban private school, UK)*

***Medical students: Why working in a PHC is a dead-end for their careers***

*"After MBBS we do not have enough knowledge, we cannot deal with patients directly. We are just beginners." (Allopathic student, 22, male, undergraduate, urban public school, AP*)

*"If a doctor goes to a PHC, today it is actually like a dead-end. There are no x-rays or ultrasound or anything. So he refers all the cases to tertiary hospitals. He only treats basic ailments." (Allopathic student, male, 23, urban public school, AP)*

*"I think recognition is one point. Generally, a PHC working doctor is considered inferior by a doctor in urban area." (MBBS student, AP)*

*"Only simple headache and fever, these can be only treated there (at a PHC). Everything else is referred to higher centers. You don't need postgraduates to do referral." (Allopathic student, 27 years, male, post graduate, rural public school, AP)*

*"If I am sitting in a rural PHCs and doing only five basic tests, then my PG knowledge is a waste." (Allopathic student, 28, female, postgraduate, urban private school, UK)*

**Nursing and Ayurvedic cadre: Jobs at PHCs aid learning**

*"In a primary health center, you'll get to know what is really going on in health. Rather than in the private sector." (Ayurvedic doctor, female, experience: 6 years, upbringing: city, AP)*

*"At PHC level nurses get opportunity to work as midwife…which is a good exposure and will help me gain experience and enable me to conduct deliveries confidently and independently." (Nurse, female, experience: 5 years, upbringing: rural, UK)*

*"Theory is different from practical—so if we doing the job we can learn more and more." (Nursing student, 20, female urban private school, AP)*
