# Supplementary material for: For more than love or money: attitudes of student and in-service health workers towards rural service in India
Source: Hum Resour Health. 2013 Nov 21;11:58. doi: 10.1186/1478-4491-11-58 (PMC4222605; doi:10.1186/1478-4491-11-58)
Supplement: Additional file 7 — Contextual factors. [file 1478-4491-11-58-S7.pdf]

Additional file 7

Contextual factors

Living facilities and transport

"Firstly better accommodation. Even basic day-to-day requirements should be there. Food and water, hygienic food…electricity. There should be good pucca (cement) house and no snakes inside the house." (Allopathic doctor, male, experience: 9.5 years, upbringing: city, AP)

"Generally, gents can move to remote places because they can go in the morning and come in the evening by vehicles. It is a problem for ladies. And in the rural areas generally they (villagers) drink. After six, they drink and they'll create some nuisance." (Ayurvedic doctor, female, experience: 12 years, upbringing: city, AP)

"We have a road to our village and one bus; sometimes it comes and sometimes it won't." (Nursing student, AP)

Children's education

'Schooling is important. Among humankind, there is a desire that offspring should do better than us." (Allopathic doctor, male, experience: 14 years, upbringing: city, AP)

"Everybody wants their children to converse in English and learn goods manners. Government schools (in rural areas) do not pass on such qualities." (Nurse, female, experience: 15 years, upbringing: small town, UK)

"(Other) students belong to poor and uneducated families…In some schools I saw teachers using bad language, in one school I saw the teacher smoking in front of the students. What will my children learn?" (Ayurvedic doctor, male, experience: 9 years, upbringing: small town, UK)

"In villages, they do not have good schools. But if I had enough money, we can send our children to good schools far off…but the children will have to go up and down every day." (Nurse, female, experience: 11 years, upbringing: village, AP)

Safety

"Sometimes I feel bad because they (village people) drink and come and fight with the sisters. They say 'We have body pain, so give us an IV,' and we cannot put in IV fluids at that moment…Already the person is drunk." (Nurse, female, experience: 10 years, upbringing: city, AP)

"A doctor in a village is insecure——if an emergency case comes and you don't have the equipment, how will you treat that patient? If anything goes wrong, then you will be surrounded by a furious crowd. I will be defamed." (Ayurvedic student, 23, female, urban private school, UK)

"Even in the government hospitals we also are not able to handle the patients who are in a drunk condition, arrogant people so…I want some people who can control this group like drunken people…here if any problem arises I will call the security outside…I will call the ward boys."
(Allopathic student, 21, female, urban public school, AP)

Social life

"*Now these days' people believe in urban lifestyle like want to live in metro cities. A medical student while studying molds his lifestyle into an urban lifestyle like movies, restaurants, etc. So they don't get adjusted there." (Ayurvedic student, 22, male, urban private school, UK)*

"*Obviously you have lot of entertainment like movies, shopping. I am a shopping freak. I even love watching movies. Everybody does. Yes definitely, maybe because I was brought up in such a manner. If you take some of my friends who were in a village maybe they won't mind losing all these things." (Allopathic student, 23, male, rural public school, AP)*

Community type

"These illiterate people…they always see you as foreigners and even if you prescribe a simple medicine and if patient died due to any other reason, the whole village will come to hold your neck."
(Allopathic student, 28, female, post graduate, urban private school, UK)

"Establishing a rapport with the villagers is difficult! You cant go settle there because you don't know them."
(Allopathic student, 23, male, urban private school, AP)
