# Supplementary material for: For more than love or money: attitudes of student and in-service health workers towards rural service in India
Source: Hum Resour Health. 2013 Nov 21;11:58. doi: 10.1186/1478-4491-11-58 (PMC4222605; doi:10.1186/1478-4491-11-58)
Supplement: Additional file 8 — Way Forward. [file 1478-4491-11-58-S8.pdf]

Additional file 8

***The way forward***

• Almost all states in India offer incentives to improve rural recruitment, but these strategies are built without taking into account the viewpoints of all relevant stakeholders. It is important to point out that incentive mechanisms may bring health workers to rural areas and fill vacant posts, but their interests may still lie elsewhere.

• The natural way forward is to explore which of the issues highlighted in this study are amenable to political reform and how the reform process can be moved forward at national and local levels.

• A disinterested, disillusioned or coerced health workforce is unlikely to foster trust in the community and be a good foundation for rural primary care. Considering this, it is critical that attention is paid to the type of clinician being posted and their motivation to live and serve in rural areas.
